# Longer Sleep Duration is Linked to More Severe Cognitive and Functional Impairments in Older African Females who are APOE‐ε4 Non‐carriers

**DOI:** 10.1002/alz70861_108958

**Published:** 2025-12-23

**Authors:** Brittany A Larsen, Debbie Chung, Joon Chung, Farid Rajabli, Azizi A Seixas, Rufus O Akinyemi, Susan H. Blanton, Michael L Cuccaro, Brian W Kunkle, Jeffery M Vance, Christiane Reitz, Giuseppe Tosto, Scott M Williams, William S Bush, Jonathan L Haines, Adesola Ogunniyi, Goldie S Byrd, African Dementia Consortium, Margaret Pericak‐Vance

**Affiliations:** ^1^ University of Miami Miller School of Medicine, Miami, FL USA; ^2^ College of Medicine, University of Ibadan, Ibadan, Oyo Nigeria; ^3^ Columbia University Irving Medical Center, New York, NY USA; ^4^ Case Western Reserve University School of Medicine, Cleveland, OH USA; ^5^ Wake Forest University School of Medicine, Winston‐Salem, NC USA

## Abstract

**Background:**

Blacks are disproportionately affected by Alzheimer’s disease (AD), with rates twice as high as Whites. Etiological factors of AD include behavioral (e.g., sleep) and biological (i.e., genetics) contributors. Both short and long sleep durations are associated with worsened cognitive/functional impairments among older adults. A high prevalence of sleep disturbances has been found in African populations. Genetically, carriers of the APOE‐ε4 allele, which are more abundant in Blacks compared to Whites, have a significantly greater risk for developing late‐onset AD compared to non‐carriers, especially for females. Although the APOE‐ε4 allele has also been linked to sleep duration in prior work, results have been inconsistent. Hence, we investigated how the relationship between sleep duration and extent of cognitive/functional impairment differed between APOE‐ε4 carriers

**Method:**

A subset of older adult participants residing throughout Africa with available APOE genotyping data (*n* =419, 51.3% female, aged 74.8±9.2 years) were included for analysis from a population‐based DAWN Alzheimer's Research Study. Subjects self‐reported sleep duration within a 24‐hour timeframe in hour increments. Each participant was genotyped and categorized as APOE‐ε4 carriers (50.4%) or non‐carriers (49.6%). The extent of cognitive/functional impairments was measured by the Clinical Dementia Rating Scale Sum of Boxes (CDR‐SB) scores. Zero‐inflated negative binomial regression tested how self‐reported sleep durations predicted CDR‐SB scores when stratified by APOE‐ε4 status and sex, controlling for age, years of education, and body mass index.

**Result:**

Across the whole sample, sleep duration was not a significant predictor of CDR‐SB scores (b=0.13, *p* >0.05), nor was there a two‐way interaction between sleep duration and APOE‐ε4 status alone (b=0.08, *p* >0.05) or sex alone (b=0.03, *p* >0.05). When stratified by APOE‐ε4 status and sex, however, longer sleep duration was associated with more cognitive/functional impairments in (b=0.58, *p* <0.01). However, this group exhibited a U‐shaped curve, with the lowest CDR‐SB scores observed among those who reported sleeping 5–7 hours before increasing for those who reported sleeping >7 hours.

**Conclusion:**

This finding suggests that changes in sleep patterns — particularly, an uncharacteristic increase in sleep duration — may be indicative of emerging/exacerbating AD‐associated pathology in older Black female APOE‐ε4 non‐carriers

